# Application of voltage controlled crystal oscillators to DC voltage reference validation

**DOI:** 10.1088/2631-8695/adc77e

**Published:** 2025-04-08

**Authors:** Matthew T Spidell, Gaylon W Partain, Alan G Jaffe, Malcom G White, John H Lehman

**Affiliations:** 1 National Institute of Standards and Technology, Boulder, CO, United States of America; 2 Army Primary Standards Laboratory, Redstone, AL, United States of America

**Keywords:** VCXO, VC/OCXO, voltage metrology, voltage reference drift, zener diode, voltage reference validation

## Abstract

DC voltage references are typically based on calibrated Zener Diodes which are subject to drift and therefore require periodic recalibration against a Primary Standard. Since it is preferable to maintain constant power to such references, the shipping and handling logistics involved in such a recalibration can present an unacceptable burden. Validation is therefore preferred, and typically accomplished by comparison against other local Zener Diodes. However, these Zener Diodes are all subject to correlated aging mechanisms. Voltage to frequency conversion represents an alternative mechanism for validation and accordingly, is not subject to aging highly correlated with the system being validated. Voltage to frequency conversion using a low space, weight, and power Voltage Controlled Oscillator offers a mechanism for identifying voltage reference drift by comparing the voltage-controlled frequency to primary frequency, typically available through GPS. This technique can validate voltage references when comparison against a primary voltage standard is impractical due to system deployment, away from a robust logistics chain. Voltage to frequency conversion may be accomplished by a Voltage Controlled Ovenized Crystal Oscillator. Using commercial-off-the-shelf hardware we constructed a test to evaluate the stability of such an oscillator for 258 days of continuous run-time, without age acceleration measures. Long-term drift was consistent with a $\sqrt{t}$ aging model. Sequestering 2/3 of the data to construct an aging model, then comparing sequestered data, yielded a model-to-data difference of 35 ppm (35 μV/V) which may prove acceptable in supporting instruments in the 6.5-digit voltmeter class.

## 1. Introduction

Precision DC Voltage Reference Standards based on metrology-grade Zener diodes can offer expanded uncertainty of 1 to 3 ppm/year [1, 2]. With multi-year trend data, they have been reported to offer uncertainty of <0.1 ppm/year [3]. For certain remote deployments and/or end users with statutory requirements, annual validation of such voltage references can be required, regardless of the acceptability of greater uncertainty [4, 5]. Therefore, fit-for-purpose validation can be accomplished with a system subject to larger drift than that provided by metrology-grade Zener diodes [4].

A typical 6.5-digit DVM may be specified with a tolerance of 75 ppm [6]. A test uncertainty ratio of 1.5:1, as indicated in ANSI Z540.3 allows us to infer that the voltage standards supporting such a typical 6.5 digit DVM would need to be validated to a level of 50 ppm or better [7, 8]. To be clear, a validation mechanism providing wider uncertainty than the DC Voltage Reference Standards serves only to support, not to replace them.

**Figure 1.**
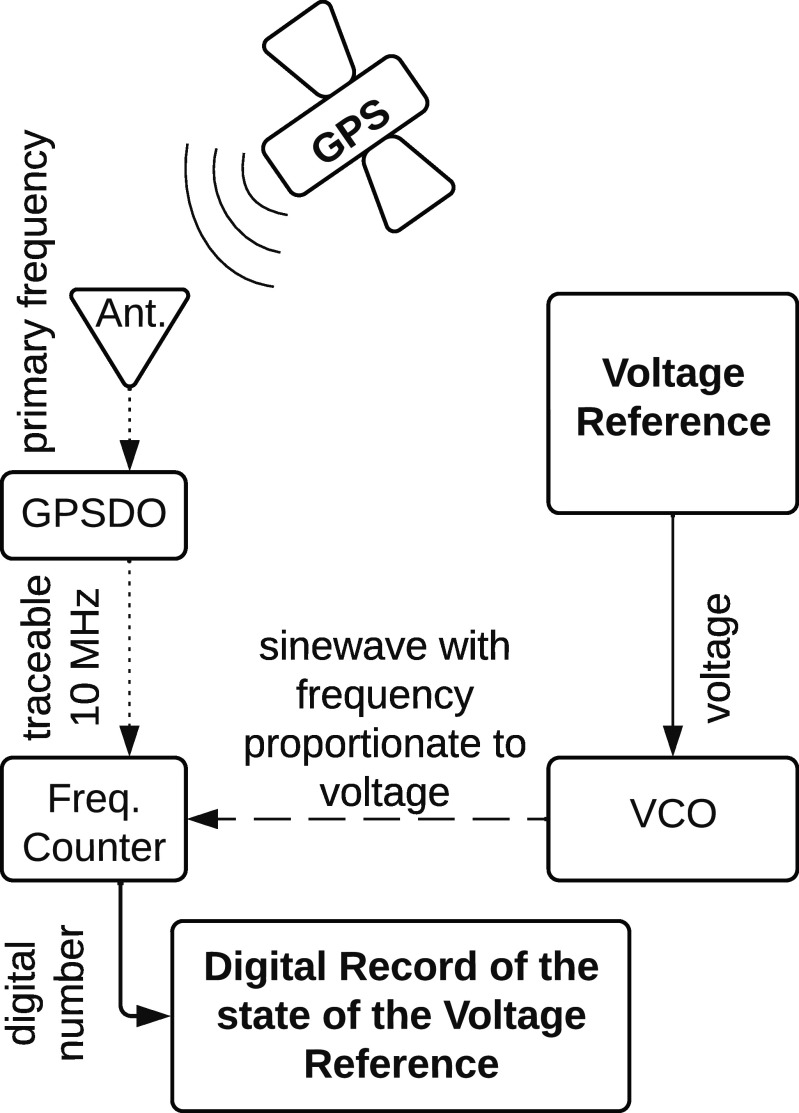
Voltage reference validation scheme diagram.

A validation scheme that offers the potential of meeting 50 ppm/yr uncertainty is a voltage to frequency conversion mechanism such as a Voltage Controlled Oscillator (VCO) paired with ubiquitous primary frequency available using a Global Positioning System (GPS) using a GPS-Disciplined Oscillator (GPSDO). An example system overview is shown in figure 1.

An electronic oscillator circuit may be constructed from a pair of components establishing a time constant (e.g. an R-C circuit) along with supporting electronics to replace losses and output a signal. A VCO circuit accepts an external control voltage to change a circuit property and thereby change the oscillation frequency [9]. Commercial-Off-The-Shelf (COTS) VCOs are available which utilize electromagnetic resonance (such as an L-C circuit), electronic discharge time constant (such as an R-C circuit), and electro-mechanical resonance (such as a Surface Acoustic Wave Oscillator or a Piezo-Varactor Oscillator) [10]. The VCO we have elected to evaluate is a Commercial-Off-The-Shelf (COTS) Voltage Controlled Ovenized Crystal Oscillator (VC/OCXO) which implements the electro-mechanical Piezo-Varactor scheme [10]. We elected to pursue this mechanism due to availability, quartz oscillator long-term stability, and direct conversion of the measurand to frequency without need of a voltage reference (other than the reference being evaluated).

## 2. Previous work

Other work studying R-C time constant VCOs suggested that an R-C system can be used to provide calibration of Precision DC Voltage Reference Standards independent of traceability to a primary standard for voltage [11]. However, this overstates what such a scheme can accomplish. In a VCO circuit constructed with conventional components (resistors, capacitors, inductors, crystals, etc) the time constant is not fixed by ‘physical constants’. Instead, the time constant depends on the construction characteristics of those components [10]. Therefore, it fails to provide a representation of voltage independent of a primary voltage standard. An initial calibration is required for VCOs constructed from conventional circuit components, contrary to the published claim.

That prior work neglected to consider that the R-C components are subject to long-term drift and fails to study such effects. Finally, if we imagine replacing the capacitor and resistor in that prior work with ’perfect’ components (constant values for resistance and capacitance) the frequency also depends on the voltage scale provided by the internal (low accuracy) Zener reference employed in such a Wein oscillator [9], which undermines the entire strategy.

## 3. Modelling VC/OCXO characteristics

An initial model to explore the feasibility of a quartz crystal VC/OCXO in this application is to treat it as a linear device that converts voltage ${\boldsymbol{V}}$ to frequency ${\boldsymbol{f}}$. The conversion has an arbitrary sensitivity of ${\boldsymbol{\alpha }}$ and an arbitrary carrier ${\boldsymbol{\beta }}$. Therefore, the model may be described by ${\boldsymbol{f}}{\boldsymbol{(}}{\boldsymbol{V}}{\boldsymbol{)}}{\boldsymbol{=}}{\boldsymbol{V}}{\boldsymbol{\alpha }}{\boldsymbol{+}}{\boldsymbol{\beta }}$.

The above expression for ${\boldsymbol{f}}{\boldsymbol{(}}{\boldsymbol{V}}{\boldsymbol{)}}$ is notional, and terms ${\boldsymbol{\alpha }}$ and ${\boldsymbol{\beta }}$ are affected by long-term drift with time ${\boldsymbol{t}}$. Therefore, ${\boldsymbol{\alpha }}$ and ${\boldsymbol{\beta }}$ are more adequately expressed as ${\boldsymbol{\alpha }}{\boldsymbol{(}}{\boldsymbol{t}}{\boldsymbol{)}}$ and ${\boldsymbol{\beta }}{\boldsymbol{(}}{\boldsymbol{t}}{\boldsymbol{)}}$. Assuming characteristics of the supporting hardware elements are optimised, long-term drift may be attributed to factors ranging from changes in mechanical parameters at the internal component mounts and electrodes to ion migration and gas adsorption [12]. A phenomenological model favored by device manufacturers for long-term drift in electronic components is $\sqrt{{\boldsymbol{t}}}$ [13]. Expressing ${\boldsymbol{\alpha }}$ with consideration of long-term drift, we describe ${\boldsymbol{\alpha }}\left({\boldsymbol{t}}\right){\boldsymbol{=}}{{\boldsymbol{\alpha }}}_{{\boldsymbol{0}}}{\boldsymbol{(}}{{\boldsymbol{\Upsilon }}}_{{\boldsymbol{\alpha }}}\sqrt{{\boldsymbol{t}}}{\boldsymbol{+}}{\boldsymbol{1}}{\boldsymbol{)}}$ (which will be referred to again as the $\sqrt{{\boldsymbol{t}}}$ aging model). Where ${{\boldsymbol{\alpha }}}_{{\boldsymbol{0}}}$ is baseline voltage to frequency sensitivity, and the ${{\boldsymbol{\Upsilon }}}_{{\boldsymbol{\alpha }}}$ is the long-term drift coefficient.

${\boldsymbol{\beta }}\left({\boldsymbol{t}}\right)$ could be described similarly to ${\boldsymbol{\alpha }}\left({\boldsymbol{t}}\right)$; however, we may entirely remove ${\boldsymbol{\beta }}\,$ drift by performing a baseline correction for every measurement of the reference voltage. This is essential because the original equipment manufacturer (OEM) for the VC/OCXO used in our experiment indicates aging of 3%/yr or 30,000 ppm/yr dominated by ${\boldsymbol{\beta }}$ drift. To remove ${\boldsymbol{\beta }}$ drift we may first measure the frequency produced by a 0 V input and then measure the frequency difference between 0 V and reference voltage. Therefore, our measurement efforts are aimed at assessing ${\boldsymbol{\alpha }}{\boldsymbol{(}}{\boldsymbol{t}}{\boldsymbol{)}}$ while ${\boldsymbol{\beta }}{\boldsymbol{(}}{\boldsymbol{t}}{\boldsymbol{)}}$ is negated.

## 4. Experimental methods

The VC/OCXO used in this study is a commercial-off-the-shelf (COTS) product based on a Stress-Compensated cut crystal. The crystal is encased in a hermetically sealed thermally stabilized package. It mates to a common CO-8 footprint and consumes approximately 4 Watts of power. The VC/OCXO device exhibited a sensitivity on order of 5 V Hz^−1^ with a carrier frequency of 10 MHz. (Crystal cut was not a selection criterion since we were limited to COTS options for this study.) The details of a typical Voltage Controlled Crystal Oscillator are described in [10].

**Figure 2.**
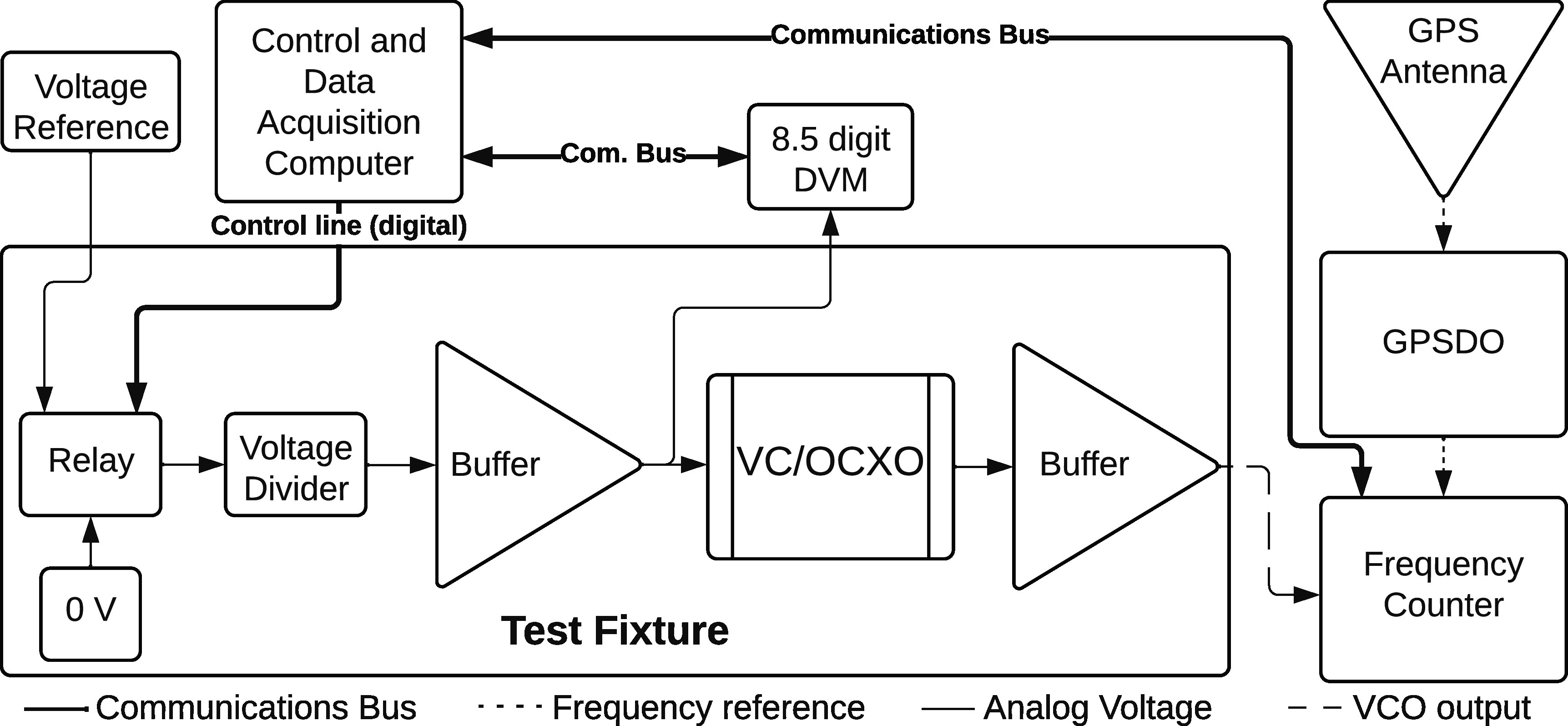
Experimental relationship of physical elements.

As shown in figure 2, the GPS antenna acquires the primary time base signal which is used by the GPSDO to steer it’s 10 MHZ oscillator. This in-turn provides a 10 MHZ frequency reference to the frequency counter, also shown in figure 2. The test fixture was a custom printed circuit board that supports components described by the block diagram elements in figure 2 which supplies the VC/OCXO with power, provides input-output buffers, and alternate the input between 0 V and the voltage reference. All components were operated at voltage and current levels within manufacturer recommendations. The test fixture was enclosed in an aluminum case and operated in a typical metrology laboratory environment of approximately 23 °C and 35% RH. Computer control supported automatic collection of monitor data and measurement data. Over 70,000 voltage-to-frequency measurements were collected over the duration of the experiment’s nearly 9-month operation.

A calibrated metrology-grade voltage reference with a 1-year tolerance of <3 ppm per the manufacturer was employed. While this voltage reference provides a nominal 10 V, this value is divided by the test fixture to 2.85 V and buffered to meet the VCO’s 3.3 V input limit and bias current draw. We will refer to this 2.85 V value again latter as ${{\boldsymbol{V}}}_{{\boldsymbol{buf}}}$. Naturally, we recognize that dividing and buffering could have introduced additional error sources, however, voltage monitoring over the duration of the experiment with a calibrated 8.5-digit DVM indicates that ${{\boldsymbol{V}}}_{{\boldsymbol{buf}}}$ drifted by less than 5 ppm. Figure 2 shows a diagram of the test fixture with supporting measurement system hardware, such as the 8.5-digit DVM monitor and voltage reference.

The voltage divider was a matched resistor network, and the buffer was a low drift unity gain amplifier. Power dissipation by the voltage divider was 3 mW, which falls well below the 125 mW limit indicated by the manufacturer. The experiment was operated from 8 June 2023 to 21 Feb 2024. The experiment observed the average frequency while applying 0 V for 100 s and again while applying ${{\boldsymbol{V}}}_{{\boldsymbol{buf}}}$. To allow for hardware delays, a measurement cycle requires 220 s. The manufacturer of the VC/OCXO indicates that minimum Allan variance of the absolute frequency occurs for an observation period of approximately 100 s, which is why we elected to use that observation period for evaluating absolute frequency. The *α* term is only observable by evaluating the difference between the frequency produced by 0 V and ${{\boldsymbol{V}}}_{{\boldsymbol{buf}}}$. We will later discuss Allan variance of the difference between the ${\boldsymbol{\alpha }}{\boldsymbol{(}}{\boldsymbol{t}}{\boldsymbol{)}}$ term and the aging model. This is not the manufacturer’s specification for Allan variance characteristics (which is dominated by the ${\boldsymbol{\beta }}{\boldsymbol{(}}{\boldsymbol{t}}{\boldsymbol{)}}$ that we are working to negate) and the reader is cautioned to avoid conflating these terms.

## 5. Results

**Figure 3.**
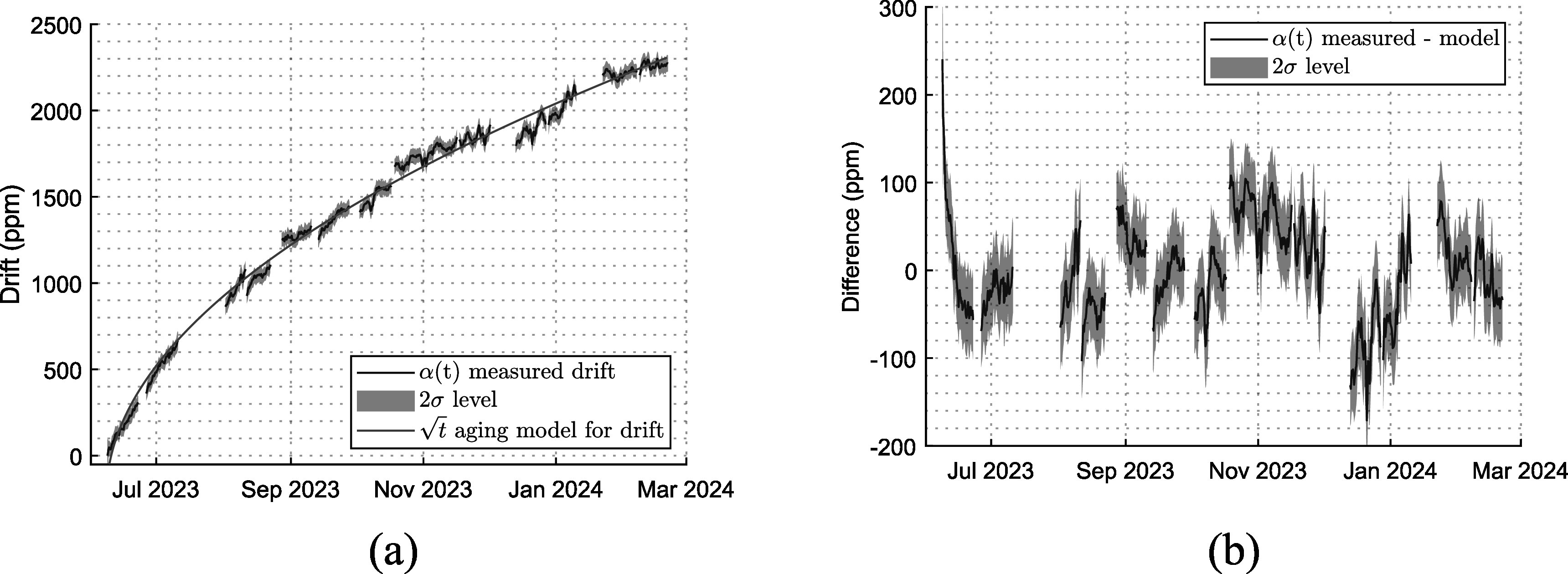
(a) Drift of the conversion factor $\alpha (t).$ Light blue depicts the 2$\sigma $ level and red depicts the aging model. (b) Difference between long-term drift of the conversion factor $\alpha (t).$ and the aging model.

In figure 3(a) the data averaged over a moving 2.5 h window is plotted in dark blue with 2 ${\boldsymbol{\sigma }}$ level for the moving 2.5 h period in light blue (the basis for this averaging period is discussed latter). It aligns with the $\sqrt{{\boldsymbol{t}}}$ model for component aging [13] plotted by the red line in figure 3(a), with fit quality of *R*^2^ = 0.997. After initial aging in the first month, greatest departures from the aging model were limited to <200 ppm as shown in figure 3(b). The breaks in the data series are attributable to interruptions in the data acquisition process owing to other demands on the facility. Errors attributable to the test fixture (cabling, buffer op-amp, and voltage divider) are limited to a total of 5 ppm by means of the 8.5-digit DVM Monitor noted earlier. Uncertainty is therefore dominated by stochastics of the measurand, not the test setup.

**Figure 4.**
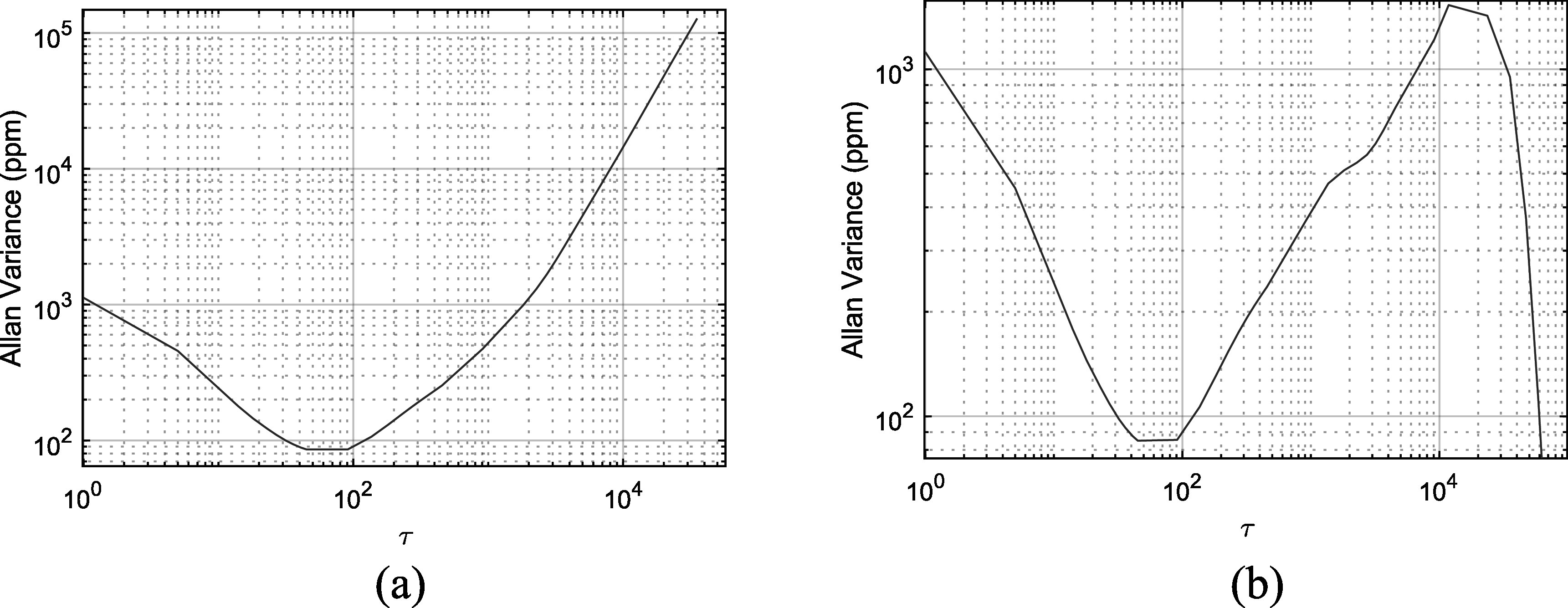
(a) Allan variance of $\alpha (t)$. (b) Allan variance of the difference between $\alpha \left(t\right)$and the aging model.

Allan variance can be used to evaluate optimum averaging time for a frequency measurement [14]. Low Allan variance is consistent with a favorable observation period. The Allan variance of ${\boldsymbol{\alpha }}{\boldsymbol{(}}{\boldsymbol{t}}{\boldsymbol{)}}$ (figure 3(a)) is shown in figure 4(a) while the Allan variance of the difference between ${\boldsymbol{\alpha }}{\boldsymbol{(}}{\boldsymbol{t}}{\boldsymbol{)}}$ and the aging model (figure 3(b)) is shown in figure 4(b). Gaps in the data were mitigated by employing the technique outlined in [15]. In figures 4(a) and (b), the ordinate axis is indicated in observation counts (${\boldsymbol{\tau }}$) which may be reinterpreted in terms of observation period using table 1. The Allan variance of ${\boldsymbol{\alpha }}{\boldsymbol{(}}{\boldsymbol{t}}{\boldsymbol{)}}$ shown in figure 4(a) is unremarkable. As one would expect for a typical oscillator, a very short observation period is subject to high Allan variance due to high short-term noise that averages out with longer observation periods. A minimum Allan variance is therefore reached for an intermediate observation period, 40 ${\boldsymbol{\tau }}\,$(roughly 2.5 h) in this case. Longer observation periods see increasing Allan variance due to the effect of aging.

**Table 1.** Observation count ($\tau $) versus observation time for notable periods applicable to figures 4(a) and (b).

Observation Time	$\tau $
220 s	1
1 h.	17
1 Day	3.9 × 10^2^
1 Week	2.8 × 10^3^
1 Month	1.2 × 10^4^

Using the $\sqrt{{\boldsymbol{t}}}$ aging model, long-term drift is predictable, as suggested by inspection of the overlap of the data (blue) and model (red), plotted in figure 3(a). Therefore, to compensate for aging, we subtracted the aging model from the aging data and plotted the result in figure 3(b). Figure 4(b) depicts the Allan variance of this aging-compensated data and like figure 4(a) shows a low Allan variance for an observation period of 40 ${\boldsymbol{\tau }}\,$ and increasing Allan variance for longer periods *up until* the periods that benefit from the long-term drift model. We therefore achieve a second drop-off, for observation periods exceeding 10^4^ ${\boldsymbol{\tau }}$, which equates to an observation duration of approximately 1 month (see table 1). This indicates that when using an aging model to compensate for drift in our system, we must use observation periods exceeding 1-month. Longer observation periods are of course preferred, though need of practical observation periods must be considered.

## 6. Analysis and discussion

Drift in the voltage to frequency conversion factor would register as drift in the voltage reference being validated in our proposed scheme. Therefore, the ideal voltage to frequency conversion mechanism would exhibit negligible long-term drift. To our knowledge, such a device is not available today.

**Figure 5.**
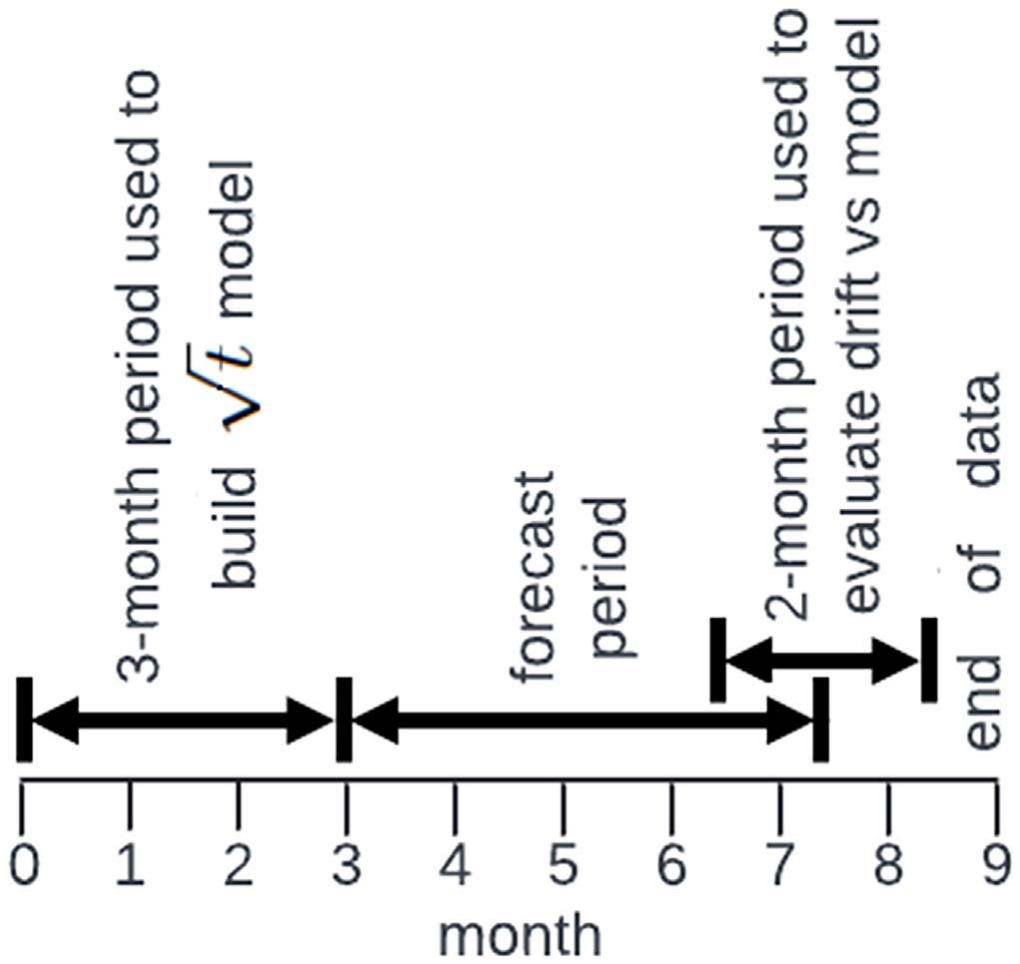
Timeline for building $\sqrt{t}$ forecast and evaluating error.

While drift is unavoidable, it can be *predictable* as evidenced by the close agreement between the $\sqrt{{\boldsymbol{t}}}$ aging model and the measured data shown in figure 3(b). Therefore, we need to establish how predictable the device performance is. Our data spans nearly 9-months. In Results, we used that entire data set to demonstrate that the $\sqrt{{\boldsymbol{t}}}$ model provided a valid description of VC/OCXO aging. Here, we sequester the last 6 months of data and use *only* the first 3 months of measured data to produce a new aging model. These time spans are illustrated in figure 5.

Since we build our $\sqrt{{\boldsymbol{t}}}$ aging model here with *only* the first three months of data, we may then compare that model with the sequestered data. This allows us to examine how well an aging model forecasts future performance when looking forward by a period approximately 1.5 × the evaluation period (see figure 5). We used a 2-month averaging period as a compromise between the benefit of a long observation period and the time range of data available. The difference between the model’s prediction (using the first three months of data to build the model) and the measured data (averaged over the final 2 months) is 35 ppm.

Contributions to aging model uncertianty include the DC Voltage reference drift, test fixture, test fixture monitor, and VC/OCXO instability. These uncertainty contributions are listed in table 2. The relative standard uncertainty contributions and the combined expanded Uncertainty (relative) were assessed per the BIPM Guide to Uncertainty [16]. The combined expanded Uncertainty is 110 ppm (relative). The 35 ppm difference between the model and measurement is modest compared to our expanded Uncertainty of 110 ppm, lending support to the uncertainty estimate.

**Table 2.** A list of uncertainty contributions.

Source	Relative standard uncertainty	Type
Reference drift	1.7 ppm	B
Test fixture	2.9 ppm	B
Monitor	4.6 ppm	B
Measurand stochastics	55 ppm	A

This data set does not represent a ‘hero’ device out of an ensemble, the results are based on the one device under test for many months. We expect that due to device-to-device production variation (subtle assembly and material differences due to processing inhomogeneity) the $\sqrt{{\boldsymbol{t}}}$ aging model coefficient for every device will differ and will need to be individually characterized.

With observation periods of two months (or longer) required to achieve acceptable uncertainty with present COTS VC/OCXO hardware, this scheme does not represent a viable alternative to existing Precision DC Voltage Reference Standards. It is only of interest for long-term validation in remote deployments or for end users with statutory requirements better met through validation than shipping hardware to a primary standards laboratory for recalibration [4, 5].

## 7. Conclusions

The success in forecasting by approximately 1.5 × the evaluation period, with a difference of 35 ppm, is a highly encouraging result given that the uncertainty required to support a 6.5-digit DVM is 50 ppm. Use of a GPSDO and a VCO in the form of a VC/OCXO to validate Zener Diode-based voltage references at a level of uncertainty sufficient to support certain end users may be achievable. Enabling shorter evaluation periods and lower stochastic contributions to uncertainty will likely require material and assembly optimization of the VC/OCXO for stability of the ${\boldsymbol{\alpha }}{\boldsymbol{(}}{\boldsymbol{t}}{\boldsymbol{)}}$ term, which was not a design criterion in existing COTS hardware.

## 8. Future work

Voltage represents a test case for other measurands that can be directly converted to frequency through electro-mechanical devices, such as piezo crystal oscillators. Since piezo crystals are sensitive to temperature and pressure, these may represent other viable candidate areas for study. The key feature in developing such schemes is conversion of the measurand to frequency without reliance on other internal representations of the same measurand. Future work must consider long-term stability of the conversion mechanism as the first line of research with functional improvements following evidence of predictable performance.

## Notes

Certain commercial equipment, instruments, or materials are identified to illustrate the relevance of the uncertainty level achieved in this effort. Such identification is not intended to imply recommendation or endorsement by NIST, nor is it intended to imply that the materials or equipment identified are necessarily the best available for the purpose. This is a work of the US Government.

## Data Availability

The data that support the findings of this study are available at https://doi.org/10.18434/mds2-3747.
